# Proteomics investigation of OSCC-specific salivary biomarkers in a Hungarian population highlights the importance of identification of population-tailored biomarkers

**DOI:** 10.1371/journal.pone.0177282

**Published:** 2017-05-18

**Authors:** Éva Csősz, Péter Lábiscsák, Gergő Kalló, Bernadett Márkus, Miklós Emri, Adrienn Szabó, Ildikó Tar, József Tőzsér, Csongor Kiss, Ildikó Márton

**Affiliations:** 1 Department of Biochemistry and Molecular Biology, Faculty of Medicine, University of Debrecen, 1. Egyetem ter, Debrecen, Hungary; 2 Department of Medical Imaging, Faculty of Medicine, University of Debrecen, 1. Egyetem ter, Debrecen, Hungary; 3 Department of Oral and Maxillofacial Surgery, Faculty of Dentistry, University of Debrecen, 1. Egyetem ter, Debrecen, Hungary; 4 Department of Periodontology, Faculty of Dentistry, University of Debrecen, 1. Egyetem ter, Debrecen, Hungary; 5 Department of Pediatrics, Faculty of Medicine, University of Debrecen, 1. Egyetem ter, Debrecen, Hungary; H Lee Moffitt Cancer Center and Research Institute, UNITED STATES

## Abstract

Oral squamous cell carcinoma (OSCC) accounting for about 90% of malignant oral lesions is the 6th most common malignancy worldwide. Diagnostic delay may contribute to dismal survival rate therefore, there is a need for developing specific and sensitive biomarkers to improve early detection. Hungarian population occupies the top places of statistics regarding OSCC incidence and mortality figures therefore, we aimed at finding potential salivary protein biomarkers suitable for the Hungarian population. In this study we investigated 14 proteins which were previously reported as significantly elevated in saliva of patients with OSCC. In case of IL-1α, IL-1β, IL-6, IL-8, TNF-α and VEGF a Luminex-based multiplex kit was utilized and the salivary concentrations were determined. In case of catalase, profilin-1, S100A9, CD59, galectin-3-bindig protein, CD44, thioredoxin and keratin-19, SRM-based targeted proteomic method was developed and the relative amount of the proteins was determined in the saliva of patients with OSCC and controls. After several rounds of optimization and using stable isotope-containing peptides, we developed an SRM-based method for rapid salivary protein detection. The validation of the selected potential biomarkers by ELISA revealed salivary protein S100A9 and IL-6 as useful protein biomarkers for OSCC detection improving the diagnostic accuracy for OSCC in the Hungarian population.A noninvasive diagnostic method to detect biomarkers useful for the early diagnosis of OSCC was developed. This can be an attractive strategy in screening saliva samples collected in a nation-wide multi-centric study in order to decrease morbidity, mortality, to enhance survival rate and to improve quality of life. The heterogeneity of protein biomarkers found in different ethnic groups presented in the literature highlights the importance of identification of population-tailored protein biomarkers.

## Introduction

With 350,000–400,000 new cases annually, oral cavity squamous cell carcinomas (OSCC) represent a major health-care problem worldwide. The increasing incidence of OSCC among women and young and middle-aged males is particularly challenging [1–4]. Despite advances in surgical and radiation treatments and chemotherapy, the average 5-year overall survival rate of advanced OSCC is not much higher than 50% [5]. Hungarian women and men occupy the top places of statistics regarding global OSCC incidence and mortality figures with an embarrassing fourfold elevation of overall OSCC mortality rate in Hungary by the new millennium since the 1970-ies [6]. In contrast to other European countries where mortality rates from OSCC started to decline after 2000, the untoward trend of rising OSCC mortality figures in Hungary did not stop [7].

Standard diagnostic methods, i.e. expert clinical examination and histological evaluation of suspicious areas fail to detect the majority of patients with early stage OSCC so improvement of the diagnostic methods is required [5,8]. Salivary protein biomarkers may represent a promising tool for improving clinical outcome of patients with OSCC.

Saliva is a very dilute body fluid which contains electrolytes, nitrogenous products and proteins. The protein content of saliva has been analyzed by several workgroups, and more than 2000 salivary proteins have been identified [9]. The most abundant proteins in saliva are α-amylase, cystatins, proline rich peptides, serum albumin and mucins [9]. Besides the highly abundant proteins, the low-abundance proteins of saliva have diagnostic values in oral diseases like bisphosphonate-related osteonecrosis of the jaw [10] and also in systemic diseases such as breast cancer [11]. In the past decade, several studies identified a series of proteins, mostly NF-κB-dependent cytokines, with elevated concentration in the saliva of patients with OSCC using antibody-based techniques, such as enzyme-linked immunosorbent assay (ELISA) as the gold standard for protein biomarker detection [12,13]. Unfortunately, this methodology does not allow the identification of protein biomarkers in great numbers, it is time consuming and expensive.

Selected reaction monitoring (SRM)-based targeted proteomics allows the identification and quantification of multiple proteins with high specificity and great detection dynamics [14]. It can be applied practically for any kind of samples but it has high importance for the study of different body fluids such as of patients with cancer [15].

Application of SRM-based targeted proteomics platform was already used for salivary protein biomarker detection [16] and our aim was to test if some of the potential salivary protein biomarkers already described in scientific literature can be used in the Hungarian population for OSCC detections.

In this prospective study we report on the detection of salivary biomarkers in accordance to the Clinical Proteomic Technologies for Cancer (CPTC) initiative guidelines established by the National Cancer Institute [17]. The guidelines suggest the application of SRM-based targeted proteomics for verification of potential biomarkers identified in the discovery phase, and after verification, the biomarkers can be subjected to the validation step. For validation, only few proteins should be chosen and tested using ELISA or other antibody-based method [17]. Candidate biomarkers reported in the literature were selected and SRM-based targeted proteomics platform along with Luminex-based multiplex assay was used to monitor the level of these biomarkers on a test set of samples followed by the verification of the potential biomarkers whose level was significantly altered in the OSCC group compared to the controls by ELISA. ELISA tests specific for the potential biomarkers were carried out on a reference set of samples and the IL-6 and S100A9 was shown to be specific for OSCC in the studied Hungarian patient cohort.

## Materials and methods

All the reagents used in this work were of analytical or LC-MS grade and were purchased from Sigma-Aldrich unless stated otherwise.

### Patients, sample collection

Unstimulated saliva samples were collected from 108 donors between 9 a.m. and 11 a.m. at the Faculty of Dentistry, University of Debrecen. Patient enrollment, sample collection and processing were carried out respecting the Declaration of Helsinki. Ethical approval was obtained from the University of Debrecen Ethics Committee (No. 3385–2011) and the subjects gave written informed consent. In parallel to sample collection a questionnaire containing questions on smoking habits, alcohol consumption was filled out by the patients. Patients were asked to avoid eating, drinking, smoking, or using oral hygiene products for at least 1 hour before sample collection.

A two-step sample collection was applied: 1) the test set (collection between 2011.06.30.- 2012.04.13.) contained 29 OSCC, 25 age-matched and 8 young controls for method development and biomarker identifications and 2) a reference set (collection between 2013.05.09–2016.02.29) containing 26 OSCC, 12 age-matched and 7 young controls for biomarker verification. Saliva samples were kept on ice throughout the collection and processing—no more than 60 minutes elapsed from sample collection to freezing. Samples of the test set were centrifuged at 4100 x g for 15 min at 4°C at the Genomic Medicine and Bioinformatics Core Facility (University of Debrecen). The supernatants were transferred to fresh tubes and the aliquots were stored at -70^°^C until further processing. The samples of the reference set were filtered using a PVDF membrane-containing filter unit (5 μm pore size, Millipore SLSV025LS) and the filtered saliva was aliquoted and stored at -70^°^C until further processing. All donors were patients of the University of Debrecen, Faculty of Dentistry. Consecutive patients with diagnosed OSCC were recruited into the study. Age-matched controls were consecutive patients admitted to the Faculty of Dentistry for regular dental checkup. The young controls were students of the University of Debrecen admitted to the Faculty of Dentistry for regular dental checkup. OSCC was diagnosed by histopathological evaluation. Treatment was started based on positive histology result and was not influenced by saliva sample collection and evaluation. Periodontal condition was evaluated by a periodontist from Department of Periodontology; none of the patients and healthy volunteers had diabetes mellitus, human papilloma virus infection or any autoimmune diseases. The study population was a consecutive series of patients and volunteers according to the above presented criteria.

### Study design

**Fig 1:**
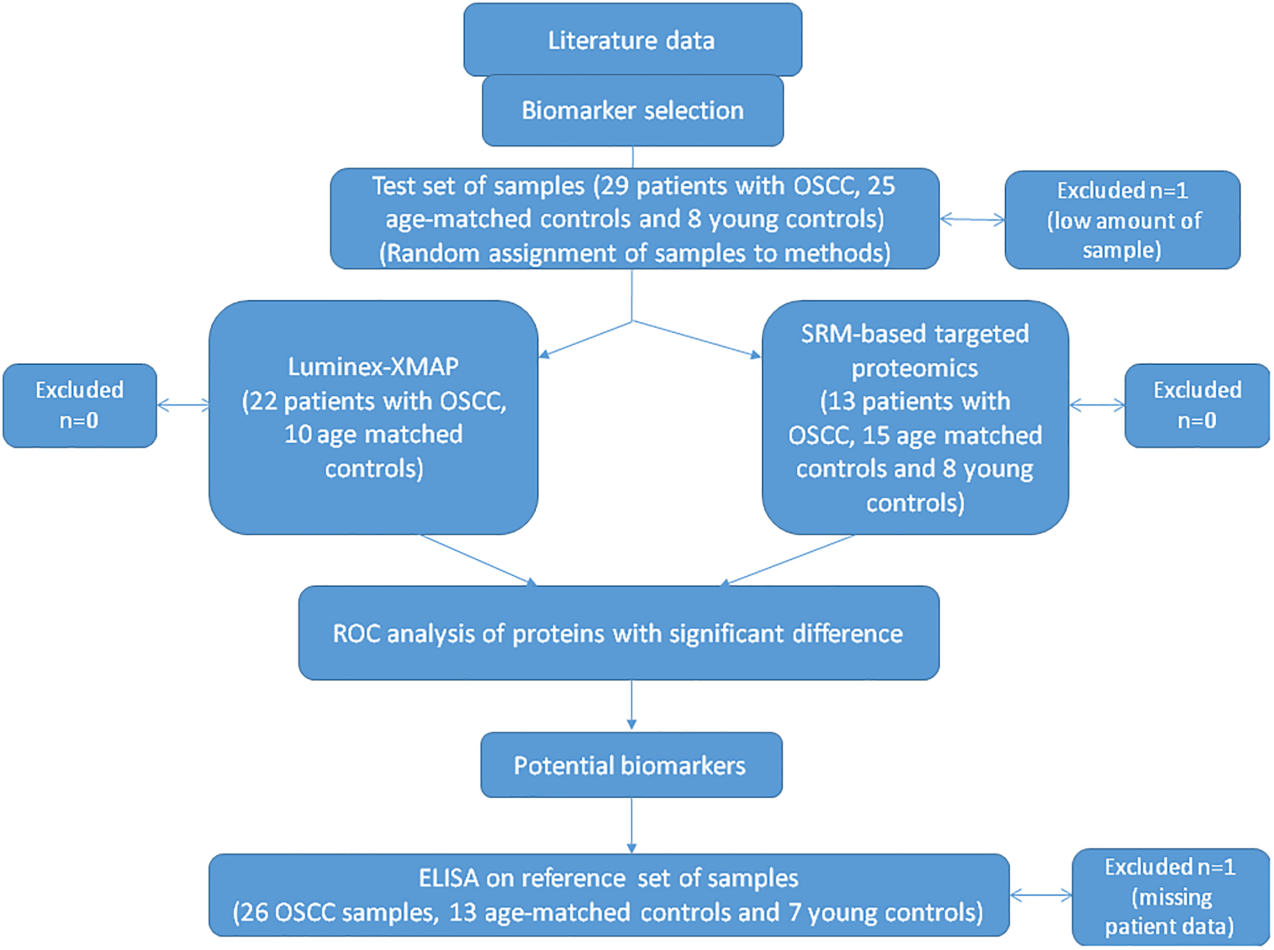
Study design.

In this prospective study we did not compare two laboratory methods rather we wanted to apply the methodology of proteomics to determine proteins with high sensitivity and specificity in saliva samples from patients with OSCC. Data collection was planned before sampling and performing the examinations. Three types of examinations were applied according to the Fig 1. During study design the CPTAC guidelines were followed: first; SRM-based biomarker verification was carried out followed by the ELISA analysis of the three selected potential biomarkers on larger patient cohort.

The samples for the Luminex assay and SRM-based assay were randomly selected from the test set of samples, and the samples for validation by ELISA were also randomly selected from the reference set of samples (S1 Table).

All the clinical evaluations of patients and controls were done by expert health care professionals (IT and AS). The laboratory examinations were done by well-trained, graduated molecular biologists (GK, PL, BM, and EC). This was a non-interventional study and the results of the performed methods did not influence in any means the treatment of patients. The sampling procedure was non-invasive and completely harmless to the study subjects. Therefore, no adverse events were related to performing the laboratory examinations.

### Cytokine assay

The multiplex immunobead Luminex x-MAP-based cytokine assay was carried out on a Custom 6plex Milliplex kit (Merck-Millipore) containing antibodies against IL-1α, IL-1β, IL-6, IL-8, TNF-α and VEGF. 25 μl of the saliva samples of patients with OSCC and age-matched controls were analyzed in duplicates. The assay was carried out based on the protocol provided by the manufacturer and the data acquisition was done on BioPlex 2.0 Workstation (Bio-Rad) operated by the Bioplex Manager 4.0 software. The level of IL-1α, IL-1β, IL-6, IL-8, TNF-α and VEGF was calculated by the Bioplex Manager software based on the recorded 7-point calibration curve. For curve fitting a logistic regression model was used.

### Design of SRM-based targeted proteomics method

The amino acid sequences of the examined proteins were utilized from the UniProt database (www.uniprot.org) and were subjected to *in silico* trypsin digestion. In order to determine the unique protein-specific tryptic sequences BLASTp analysis (http://blast.ncbi.nlm.nih.gov) was carried out searching the NCBI non-redundant protein sequence database. SRM transition design was performed by the Skyline software [18] (www.brendanx-uw1.gs.washington.edu) on the protein-specific tryptic peptide sequences. All possible transitions of singly charged “y” ions were tested on digested saliva samples from patients suffering from OSCC. Peptides which gave reproducible SRM spectra with good peak shape were chosen for further analyses and their stable isotope-labeled synthetic forms were obtained from the JPT Peptide Technologies GmbH, Germany. The quality of the synthetic peptides was assessed in our laboratory by mass spectrometry analyzes. The SRM spectra of all fragment ions were recorded and the two best transitions were selected for further analyzes.

### Sample preparation for mass spectrometry

200 μl filtered saliva was dried in speedvac and redissolved in 50 mM ammonium bicarbonate buffer. Protein concentration of the samples was determined using the Bradford method [19]. Sample blocking was carried out before trypsin digestion; one randomly selected OSCC sample was grouped with one randomly selected age-matched and one young control sample and the groups were processed together on the same day. The proteins were denatured with 6 M urea and then reduced with 10 mM dithiothreitol. The samples were alkylated with 20 mM iodo-acetamide and diluted with 25 mM ammonium bicarbonate in order to decrease the urea concentration to 1 M. Trypsin digestion was performed at 37^°^C overnight using MS grade modified trypsin (ABSciex) in 1:25 enzyme to protein ratio. The digested samples were dried in speedvac and redissolved in 1% formic acid. The samples were desalted using Pierce C18 Tips (Thermo Scientific) and the eluates were dried and dissolved in 1% formic acid.

### Mass spectrometry analysis

SRM-based analysis of saliva samples were carried out on a 4000 QTRAP (ABSciex) mass spectrometer using a NanoSpray II MicroIon Source and controlled by the Analyst 1.4.2 software (ABSciex). The spray voltage was 2800V, the ion source gas was 50 psi, the curtain gas was 20 psi and the source temperature was 70^°^C. The dwell time was 20 msec and the cycle time was 1.7 sec allowing the collection of approximately 15 data points/chromatographic peak. The chromatographic separation was done on an EasynLC II system (Bruker) and the peptide mixture was first loaded and desalted onto an in-line trap column (5 x 0.3mm, 5μm particle size, 300 Å pore size Zorbax 300SB-C18,) followed by separation on a Zorbax 300SB-C18 analytical column (150 mm x 75μm 3.5μm particle size, 300 Å pore size) using a 90 min acetonitrile/water gradient with a slow increase in acetonitrile concentration from 0% to 100% during 60 min. Solvent A was 0.1% formic acid in LC water, solvent B was LC acetonitrile containing 0.1% formic acid.

For SRM analysis 20 μg digested protein spiked with the stable isotope-labeled reference peptides was introduced to the mass spectrometer. All SRM analyses were carried out in duplicates.

### ELISA

Each saliva sample from patients with OSCC, matched control and young control subjects were analyzed in duplicate by ELISA using Human ELISA Kit. The concentration of IL-6 and thioredoxin (EK0410 and EK1254, respectively, Boster Biological Technology Co., Pleasanton, USA) and S100A9 (E-EL-H1290, Elabscience Biotechnology Co., Wuhan, China) in saliva were determined by sandwich enzyme-linked immune-sorbent assay technique according to manufacturer’s protocol. Absorbance was measured at 450 nm and concentrations were calculated based on the recorded 6-point (thioredoxin and S100A9) and 7-point (IL-6) calibration curves, respectively.

### Data analysis

The SRM data were analyzed using the Skyline software [18]. All data where the AUC value was less than 10 were excluded from further analyses. The Skyline data are publicly available through the Panorama [20] web site:

(https://panoramaweb.org/labkey/project/University%20of%20Debrecen/OSCC%20saliva/begin.view?)

The primary data were transformed into appropriate format of MSstats R-package [21–23] by an in-house developed software. Before the statistical analysis peptides with insufficient amount of data across the set of samples were removed and for further analyses only those samples were used where both technical replicates were recorded. After normalization based on heavy standards and log2 transformation of measured abundances the group differences were investigated by mixed-effect variance analysis [22,24] for all proteins. The groups were used as the fixed effect and the subject level variance were modeled as random effects. The raw p-values of group differences were adjusted by the Benjamini and Hochberg type false discovery rate (FDR) method for multiple testing issues [25]. Besides the adjusted p-values the log2 fold changes, standard error and T values were examined. For evaluation of test performances multivariate receiver operating characteristic (ROC) curve analyses [26] were constructed by the Epi R-package [27], the accuracy and the 95% confidence intervals were calculated.

## Results and discussion

Detection of salivary biomarkers which can help in the early diagnosis of OSCC is of high importance in improving survival rates of patients with OSCC. More than 35 proteins were identified by different groups as potential salivary biomarkers but a high variability in the results was observed [28]. Protein biomarkers identified in one population may not necessarily be suitable in another population. For example, IL-8 was identified as a biomarker in the USA [12,29–32], India [13] and Serbia [33] but not in Japan and Iran [34,35]. Similarly, S100A9 proved to be a suitable OSCC biomarker in the USA but not in China [36,37]. In USA catalase was shown to be biomarker by Hu et. al. [36] but not by de Jong et. al. [38]. The difference of the useful biomarkers studied in different populations (Table 1) raises the question if biomarkers validated in one population have predictive value in another population [12,29–37,39–50,38,51–60]. The prevalence of OSCC in Hungary is the highest in Europe so our aim was to examine if some of the previously described OSCC biomarkers can be used in the Hungarian population.

**Table 1:** List of examined salivary protein biomarkers for OSCC in different countries. The proteins included in the studies carried out in different countries are indicated. The proteins in bold were found to be potential biomarkers for OSCC. The abbreviations of protein names are used according to gene name.

Country	Examined proteins	Reference
Brazil	**C1R, C3, C4B, CFB, FAM49B, LCN2, LRG1, SERPINA1, SLPI, TAGLN2**	[16]
China	**CEACAM5, S100A7, S100A8**, S100A9	[37,59]
Croatia	**FGF2, IL6**	[51]
Hungary	CAT, CD44, CD59, IL1A, IL1B, **IL6,** IL8, KRT19, **LGALS3BP**, PROF1, **S100A9**, TNF, TXN, VEGF	present study
India	LDH**, MUC16, IL-8	[13,58,64]
Iran	IL1A, **IL6**, IL8, TNF	[35]
Israel	**ALB, CEACAM5**, **CCNDBP1, KRT19,** EGFR, **IGG, IGF1, LDH, MKI67**, **MMP2, MMP9, MUC16, OGG1, SRC, SERPINB5**, sIgA**, SERPINB3, TPA***	[54–56]
Italy	**STATH, BIRC5**	[52,53]
Japan	**DEFA1, IL1B**, **IL6,** IL8, SPP1	[34,39,40]
Serbia	**IL1B, IL8, M2BP**	[33]
Taiwan	ALB, AMY**, ANXA2, APOA1, APOA2, APOA4, C4BPA, C6, C9, CCNDBP1, CD109, CHIT1, **EGFR, EPHX1, FSCN1, FABP, GSTM1, HEP2, HEXA, HIST1H2AA, HPRT, HSD17B4, ICAM3, IGHA2, IGHV1-2, IGKV1D-12, IGKV4-1, IGLV1-47, ITIH2, ITIH4, NKIRAS1, KNG1, PRCP, RETN, RPL7, S100A8, SERPINA6, SLC4A1, TF, TMEM132A, TUBA1C, VTN, ZN497, ZN501**	[42–45]
United Kingdom	**P53ab**	[41]
USA	ACT**, FGF2, CAT*, CD44, CD59, EDN1, H1F0, **IL1A, IL1B, IL6, IL8**, INL, KRT19, **LGALS3BP**, MSN, MYL**, PROF1, RAB7A, S100A12, S100A7, **S100A9,** S100P, **TNF, VEGFA**	[29–32,36,46–50,38,65]

*In case of catalase protein there are controversial data; in the study carried out by Hu et. al. [36] it was reported as potential biomarker but the study carried out by de Jong et. al. [38] could not confirm this finding.

** Specific isoforms are undistinguishable.

*** Mixture of fragments of cytokeratins 8, 18 and 19.

ACT: actin, ALB: serum albumin, AMY: α-amylase, ANXA2: annexin A2, APOA1: apolipoprotein A1, APOA2: apolipoprotein A2, APOA4: apolipoprotein A–IV, BIRC5: Baculoviral IAP repeat-containing protein 5, C1R: complement C1r subcomponent, C4B: complement C4-B, C4BPA: C4b-binding protein α chain, C3: complement C3, C6: complement C6, C9: complement C9, CAT: catalase, CCNDBP1: cyclin D1-binding protein 1, CD44: CD44 antigen, CD59: CD59 antigen, CD109: CD109 antigen, CEACAM5: carcinoembryonic antigen-related cell adhesion molecule 5, CFB: complement factor B, CHIT1: chitotriosidase-1, DEFA1: human neutrophil defensin 1, EGFR: epidermal growth factor receptor, EDN1: endothelin-1, EPHX1: epoxide hydrolase 1, FABP4: fatty acid-binding protein adipocyte, FAM49: protein FAM49B, FGF2: basic fibroblast growth factor 2, FSCN1: fascin, GSTM1: glutathione S-transferase Mu1, H1F0: histone H1.0, HEP2: heparin cofactor 2, HEXA: β-hexosaminidase subunit α, HIST1H2AA: histone H2A type 1-A, HPRT: haptoglobin-related protein, HSD17B4: peroxisomal multifunctional enzyme type 2, ICAM3: intercellular adhesion molecule 3, IGF1: insulin-like growth factor I, IGG: immunoglobulin G, IGHA2: immunoglobulin alpha-2 chain C region, IGHV1-2: immunoglobulin heavy variable 1–2, IGKV1D-12: immunoglobulin kappa variable 1D-12, IGKV4-1: immunoglobulin kappa variable 4–1, IGLV1-47: immunoglobulin lambda variable 1–47, IL1A: interleukin-1α, IL1B: interleukin-1β, IL6: interleukin-6, IL8: interleukin-8, INL: involucrin, ITIH2: inter-α-trypsin inhibitor heavy chain H2, ITIH4: inter-α-trypsin inhibitor heavy chain H4, KNG1: kininogen-1, KRT19: cytokeratin-19, LCN2: neutrophil gelatinase-associated lipocalin, LDH: lactate dehydrogenase, LGALS3BP: galectin-3-binding protein, LRG1: leucine-rich alpha-2-glycoprotein, MKI67: proliferation marker protein Ki-67, MMP2: matrix metalloproteinase 2, MMP9: matrix metalloproteinase 9, MSN: moesin, MUC16: cancer antigen 125, MYL: myosin regulatory light polypeptide, NKIRAS1: NF-kappa-B inhibitor-interacting Ras-like protein 1, OGG1: N-glycosylase/DNA lyase, SPP1: osteopontin, SRC: Proto-oncogene tyrosine-protein kinase Src, P53ab: p53 autoantibody, PRCP: lysosomal Pro-X carboxypeptidase, PROF1: profiling 1, RAB7A: Ras-related protein Rab-7a, RETN: resistin, RPL7: 60S ribosomal protein L7, S100A7: protein S100-A7, S100A8: protein S100-A8, S100A9: protein S100-A9, S100P: protein S100-P, SERPINA1: alpha-1-antitrypsin, SERPINA6: corticosteroid binding globulin, SERPINB3: serpin B3, SERPINB5: serpin B5, sIgA: secreted immunoglobulin A, SLC4A1: band 3 anion transporter, SLPI: antileukoproteinase, STATH: statherin, TAGLN2: transgelin-2, TF: serotransferrin, TMEM132A: transmembrane protein 132A, TNF: tumor necrosis factor, TUBA1C: tubulin-α-1C chain, TPA: tissue polypeptide antigen, TXN: thioredoxin, VEGFA: vascular endothelial growth factor A, VTN: vitronectin, ZN497: zinc finger protein 497, ZN501: zinc finger protein 501.

Based on literature data, 14 proteins were chosen and their levels were examined in the saliva of patients with OSCC and controls originating from the North-Eastern part of Hungary. Oral hygiene of age-matched controls was quite poor, characterized by the presence of various oral inflammatory conditions, independently from the presence of OSCC, as expected from earlier stomatoepidemiologic studies investigating this population [61,62]. Therefore, recruitment of healthy young controls with good oral hygiene, absent from oral inflammatory lesions was considered important.

Multiplex analyses were carried out; the level of IL-1α, IL-1β, IL-6, IL-8, TNF-α and VEGF was examined using a custom Luminex assay taking the benefit of high sensitivity of the antibody-based methods. We have chosen these cytokines as far as their levels were observed to be elevated in saliva from patients with OSCC compared to the levels observed in controls [12,29–31,33–35,40,46]. However, in some other studies no significant changes could be observed in the levels of IL-6 and IL-8 [29,34,35]. In order to examine the level of catalase, profilin-1, S100A9, CD59, galectin-3-bindig protein, CD44 and keratin-19 identified as biomarkers by different groups [36,50,54,63], targeted proteomics method was developed and optimized.

After careful examination of the recorded metadata originating from each patient, a considerable difference regarding the alcohol consumptions and smoking habits between the groups was observed. Smoking was more abundant in patients with OSCC; they consumed more alcohol and more of them consumed alcohol on a daily base. Accordingly, the cariological and periodontal status of patients was poor; the DMFT, DMFS, gingival index and plaque index values were higher in the OSCC group compared to controls (Table 2).

**Table 2:** Patient data.

	Test set					Reference set		
	OSCC patients (BioPlex)	Age-matched control patients (Bioplex)	OSCC patients (mass spectrometry)	Age-matched control patients (MC—mass spectrometry)	Young healthy control patients (HC—mass spectrometry)	OSCC patients (ELISA)	Age-matched control patients (MC-ELISA)	Young healthy control patients (YC-ELISA)
**Total number**	22	10	13	15	8	26	12	7
**Age**	61.9±7.8 (49–77)	63.5±6.7 (53–73)	60.8±7.5 (52–77)	64.9±10.4 (52–87)	24.3±1.9 (22–28)	58.2±9.7 (44–77)	59.3± 5.6 (50–68)	24.4± 1.3 (22–26)
**Sex M/F**	18/4	9/1	11/2	11/4	3/5	20/6	3/9	4/3
**Socioeconomical status**								
**· low/middle/ high**	6/14/0	2/6/2	4/7/0	0/10/5	0/0/8	7/17/1	2/8/0	0/0/5
**Smoking habit**								
**· Never smoker**	5%	40%	7.7%	79%	62.5%	4%	41%	43%
**· Ex-smoker**	22%	10%	23%	21%	12.5%	23%	25%	14%
**· Current smoker**	68%	50%	61.6%	-	12.5%	69%	17%	14%
**· No response**	5%	-	7.7%	-	12.5%	4%	17%	29%
**Alcohol consumption**								
**· Never/rarely**	2/3	2/1	1/0	4/3	0/3	4/6	4/2	0/3
**· Monthly/ weekly**	4/4	4/3	4/1	3/3	3/1	3/8	0/4	2/0
**· Daily**	8	-	6	0	0	4	0	0
**· Intake (mL/day)**	beer 285 mL/day/person	beer 171 mL/day/person	beer 426 mL/day/person	beer 30 mL/day/person	beer 33 mL/day/person	beer 162 mL/day/person	beer 171 mL/day/person	beer 0 mL/day/person
	wine / champagne 94 mL/day/person	wine / champagne 34 mL/day/person	wine / champagne 130 mL/day/person	wine / champagne 11 mL/day/person	wine / champagne 11 mL/day/person	wine / champagne 84 mL/day/person	wine / champagne 31 mL/day/person	wine / champagne 23 mL/day/person
	spirits 25 mL/day/person	spirits 0 mL/day/person	spirits 28 mL/day/person	spirits 9 mL/day/person	spirits 10 mL/day/person	spirits 24 mL/day/person	spirits 0 mL/day/person	spirits 0 mL/day/person
**Periodontal assessment**								
**· Gingival index**	0.7	0.3	0.9	0.5	0.5	0.7	0.2	0.3
**· Plaque index**	1.4	0.4	1.6	0.3	0.2	1.3	0.6	0.3
**Cariological assessment**								
**· DMFT**	23.1	19.7	23	18.9	7.5	26.9	26	8.3
**· DMFS**	99.8	81.6	67.5	79.9	17.5	121.6	117.8	18.0
**Comorbidities**								
**·Hypertonia+Cardiovascular disease**	1	2	1	1	-	6	2	-
**· Hypertonia**	4	2	4	4	-	8	3	-
**· Cardiovascular disease**	6	0	4	0	-	2		-

### Antibody-based analysis of potential OSCC salivary biomarkers

Investigating salivary appearance of proinflammatory cytokines in OSCC patients is relevant and clinically meaningful. Cancer and chronic inflammation are interrelated with a link represented by cytokines and chemokines [66]. Both tumor cells and tumor-infiltrating immune cells produce these regulatory compounds offering thereby multiple therapeutic targets for immunomodulatory treatment of patients with oral and head and neck squamous cell carcinoma [67–69]. However, the source of the investigated cytokines in the saliva of patients with chronic oral inflammation, independent from OSCC, can be from immune cells and stimulated gingival and oral mucosal tissue [70].

**Fig 2:**
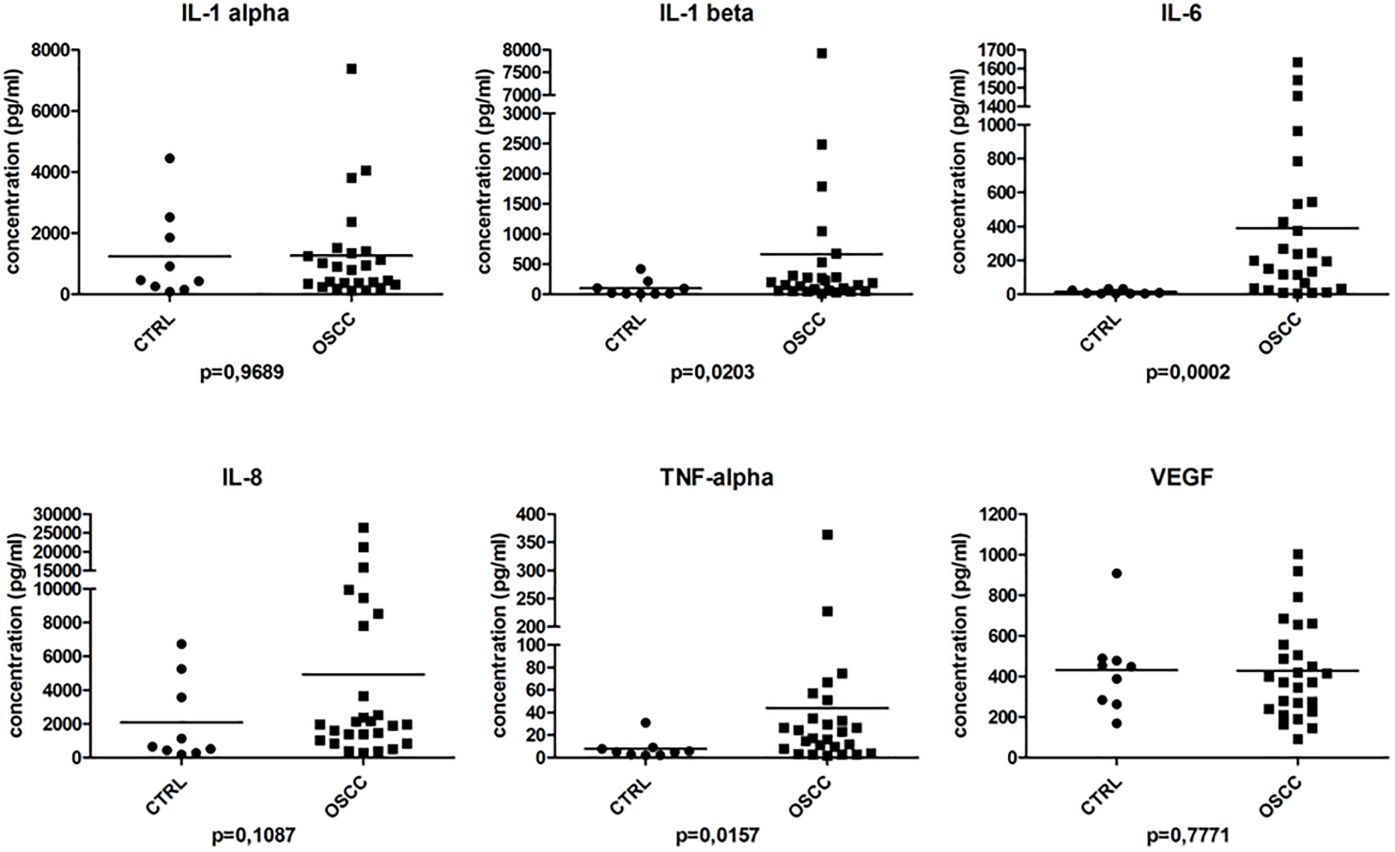
Salivary cytokine levels determined with Luminex-based multiplex assay. The concentration (pg/ml) of the studied cytokines is indicated in case of control (CTRL) and OSCC group. Below each graph the p value is indicated.

The saliva samples of randomly selected 26 patients and 9 age-matched controls were analyzed in duplicates and the levels of IL-1α, IL-1β, IL-6, IL-8, TNF-α and VEGF was determined (Fig 2). In our study the levels of IL-1β, IL-6 and TNF-α were significantly elevated in the OSCC group compared to the age-matched controls being in accordance with the previous results reported by different groups [28,33]. Examining the distribution of data points, it was observed that the results for IL-1β in the lower concentration range overlapped between the OSCC and control groups and despite the significant difference they could not discriminate between the two groups. Based on our results, it seems that among the studied cytokines only IL-6 and TNF-α can be used as potential biomarkers in the Hungarian population.

IL-6 is expressed by OSCC tumor cells and stromal cells and it has been shown to play a crucial role in OSCC carcinogenesis, progression and recurrence involving the IL-6, IL-6R, STAT3 pathway [71–73]. Using a different signal transduction pathway resulting in NF-κB activation, TNF-α has also been related to oral carcinogenesis [74]. Factors, known to be associated with poor oral hygiene and oral inflammation, such as advanced age and smoking have been shown to correlate with elevated salivary IL-6 levels [75]. Therefore, not every cytokine may serve as a suitable diagnostic salivary biomarker of OSCC in different populations.

The age-matched controls did not show any signs of precancerous lesions in their oral cavity but as a result of oral inflammatory conditions we could not see any significant differences in the levels of other cytokines between the OSCC and control groups. These results might be explained by the fact that oral hygiene in the age matched group was compromised resulting in inflammation without any signs or symptoms of OSCC. Cheng and coworkers have demonstrated that the levels of IL-6 and IL-8 were significantly higher in the saliva of patients with OSCC compared to those who have chronic periodontitis [46]. Our results confirmed these findings. IL-6 level proved to be significantly higher in patients with OSCC than in controls exhibiting a compromised oral health condition and the same trend was true in case of IL-8 (Fig 2). OSCC patients formed two subgroups with respect to salivary IL-8 concentration, 7 patients had above-average and 19 patients had below average IL-8 levels. Similar to IL-1β, IL-8 level in the lower concentration range overlapped between the OSCC and control groups. Although salivary levels of IL-8 tended to be higher in OSCC patients than in age-matched controls, the difference in this cohort was not significant. A similar dual distribution of serum IL-8 concentration and IL-8 expression by the tumor cells in patients with OSCC was observed recently by Fujita et al. High serum IL-8 concentrations, and intensive IL-8 expression by tumor cells were significantly correlated with poor disease outcome measures [76].

### Design of targeted proteomics method

An SRM-based targeted mass spectrometry method was designed for the examination of catalase, profilin-1, thioredoxin, protein S100A9, CD59 glycoprotein, CD44 antigen, galectin-3-bindig protein and keratin-19. The sequences were retrieved from the Uniprot database (P04040, P07737, Q99757, P06702, P13987, P16070, Q08380, and P08727, respectively) and *in silico* digested with trypsin. The tryptic peptides were searched against the NCBInr database using the Blastp software in order to identify the peptides which are specific for the studied proteins. Using the protein-specific sequences SRM transitions have been designed with the help of the Skyline software. The stabile isotope-labeled (SIL) synthetic counterparts of the specific peptides were ordered and spiked into the samples in known amounts. For collision energy (CE) and declustering potential (DP) optimization the synthetic peptides were used. All the possible transitions of singly charged “y” ions were optimized with the help of the Skyline software and the best two transitions per peptide were selected. The optimized parameters (Table 3) were used for the SRM method development and further used for the examination of saliva samples.

**Table 3:** Optimized SRM parameters used for the mass spectrometric examination of salivary proteins. The Q1 and Q3 m/z values, the type of “y” ion used, the dwell time, declustering potential (DP) and collision energy (CE) is indicated in case of each studied peptide.

Protein	Peptide	Q1 (m/z)	Q3 (m/z)	Ion	Dwell time (msec)	DP	CE
Catalase	LNVITVGPR	484.798	642.393	y6	20	63.0	23.4
		484.798	529.309	y5	20	63.0	23.4
		484.798	428.261	y4	20	63.0	23.4
	LNVITVGPR*	489.802	652.401	y6	20	63.0	23.4
		489.802	539.317	y5	20	84.8	23.4
		489.802	438.269	y4	20	84.8	23.4
CD44 antigen	NDVTGGR	359.677	489.277	y5	20	84.8	16.2
		359.677	390.209	y4	20	84.8	16.2
	NDVTGGR*	364.681	499.286	y5	20	84.8	16.2
		364.681	400.217	y4	20	84.8	16.2
	TEAADLCK	425.702	620.307	y6	20	63.1	20.0
		425.702	549.270	y5	20	63.1	20.0
		425.702	478.232	y4	20	63.1	20.0
	TEAADLCK*	429.709	628.321	y6	20	63.1	20.0
		429.709	557.284	y5	20	66.6	20.0
		429.709	486.247	y4	20	66.6	20.0
CD59 glycoprotein	AGLQVYNK	446.747	651.346	y5	20	66.6	21.2
		446.747	523.287	y4	20	66.6	21.2
	AGLQVYNK*	450.755	659.360	y5	20	66.6	21.2
		450.755	531.301	y4	20	66.6	21.2
Keratin-19	APSIHGGSGGR	498.254	627.295	y7	20	79.9	24.1
		498.254	490.236	y6	20	79.9	24.1
		498.254	433.215	y5	20	79.9	24.1
		498.254	376.193	y4	20	79.9	24.1
	APSIHGGSGGR*	503.258	637.304	y7	20	64.3	24.1
		503.258	500.245	y6	20	64.3	24.1
		503.258	443.223	y5	20	64.3	24.1
		503.258	386.202	y4	20	64.3	24.1
Profilin-1	TLVLLMGK	437.775	660.411	y6	20	60.6	20.7
		437.775	561.342	y5	20	60.6	20.7
		437.775	448.258	y4	20	60.6	20.7
	TLVLLMGK*	441.782	668.425	y6	20	60.6	20.7
		441.782	569.357	y5	20	63.0	20.7
		441.782	456.273	y4	20	63.0	20.7
	SSFYVNGLTLGGQK	735.882	887.494	y9	20	63.0	37.7
		735.882	773.451	y8	20	63.0	37.7
		735.882	603.346	y6	20	84.8	37.7
	SSFYVNGLTLGGQK*	739.890	895.508	y9	20	84.8	37.7
		739.890	781.465	y8	20	84.8	37.7
		739.890	611.360	y6	20	84.8	37.7
Protein S100A9	DLQNFLK	439.242	649.366	y5	20	84.8	20.8
		439.242	521.308	y4	20	84.8	20.8
	DLQNFLK*	443.249	657.380	y5	20	63.1	20.8
		443.249	529.322	y4	20	63.1	20.8
	LTWASHEK	486.250	757.362	y6	20	63.1	23.5
		486.250	571.283	y5	20	63.1	23.5
		486.250	500.246	y4	20	66.6	23.5
	LTWASHEK*	490.257	765.376	y6	20	66.6	23.5
		490.257	579.297	y5	20	66.6	23.5
		490.257	508.260	y4	20	66.6	23.5
Thioredoxin	TAFQEALDAAGDK	668.822	760.383	y8	20	66.6	33.9
		668.822	689.346	y7	20	66.6	33.9
	TAFQEALDAAGDK*	672.829	768.397	y8	20	79.9	33.9
		672.829	697.360	y7	20	79.9	33.9
	VGEFSGANK	454.727	623.314	y6	20	79.9	21.7
		454.727	476.246	y5	20	79.9	21.7
	VGEFSGANK*	458.734	631.328	y6	20	64.3	21.7
		458.734	484.260	y5	20	64.3	21.7
Galectin-3-binding protein	LASAYGAR	404.719	624.310	y6	20	64.3	18.8
		404.719	466.240	y4	20	64.3	18.8
	LASAYGAR*	409.723	634.318	y6	20	60.6	18.8
		409.723	476.249	y4	20	60.6	18.8

* indicates the stable isotope-labeled, heavy amino acid.

In order to gain information regarding the concentration range where the peptide amount introduced into the mass spectrometer is linear with the intensity of the signal recorded, a dilution series of approximately 60 fmol-1 pmol of SIL peptides spiked into saliva samples were analyzed. For keratin-19 and CD44 antigen peptides no linearity could be observed. For galectin 3 binding protein LASAYGAR, protein S100A9 LTWASHEK, thioredoxin VGEFSGANK and profilin-1 TLVLLMGK peptides the curve was linear between approximately 120 fmol-0.5 pmol range, while in case of catalase LNVITVGPR, CD59 glycoprotein AGLWVYNK peptides the linear range was slightly broader; between approximately 120 fmol-1 pmol. The thioredoxin TAFQEALDAAGDK peptide shows linearity between approximately 60 fmol-0.5 pmol concentrations, while the whole studied range was covered only by protein S100A9 DLQNFLK peptide (approximately 60 fmol-1 pmol concentrations). The broad concentration range observed permits the study of high-amplitude changes often present in biological systems.

### Targeted proteomic analysis of the selected potential OSCC biomarkers

The SRM transitions for endogenous peptides and SIL counterparts were recorded and using the amount of the SIL peptide as a reference the relative levels of endogenous peptides were calculated. In order to assess which peptides and which transitions can be successfully utilized in an eventual screening, saliva samples of randomly selected 15 patients with OSCC, 15 age-matched controls (MC) and 10 healthy young controls (HC) were analyzed using the developed targeted mass spectrometry method. In all cases where the area under the curve values for the transitions were less than 10, the results were omitted and not used for further calculations.

Some of the transitions did not give consistent results in the analyzed samples so they were omitted from the calculations. This means that for CD44 antigen, CD59 glycoprotein, galectin-3-bindig protein and keratin-19 the best transitions used during the optimization did not provide good quality data in most of the analyzed saliva samples so they were not used for the statistical analysis. The catalase LNVITVGPR peptide, the SSFYVNGLTLGGYK and TLVLLMGK peptides of profilin-1, the DLYNFLK peptide from protein S100A9 and TAFQEALDAAGDK peptide from thioredoxin gave data of acceptable quality in most of the samples and were further used for statistical analysis.

**Fig 3:**
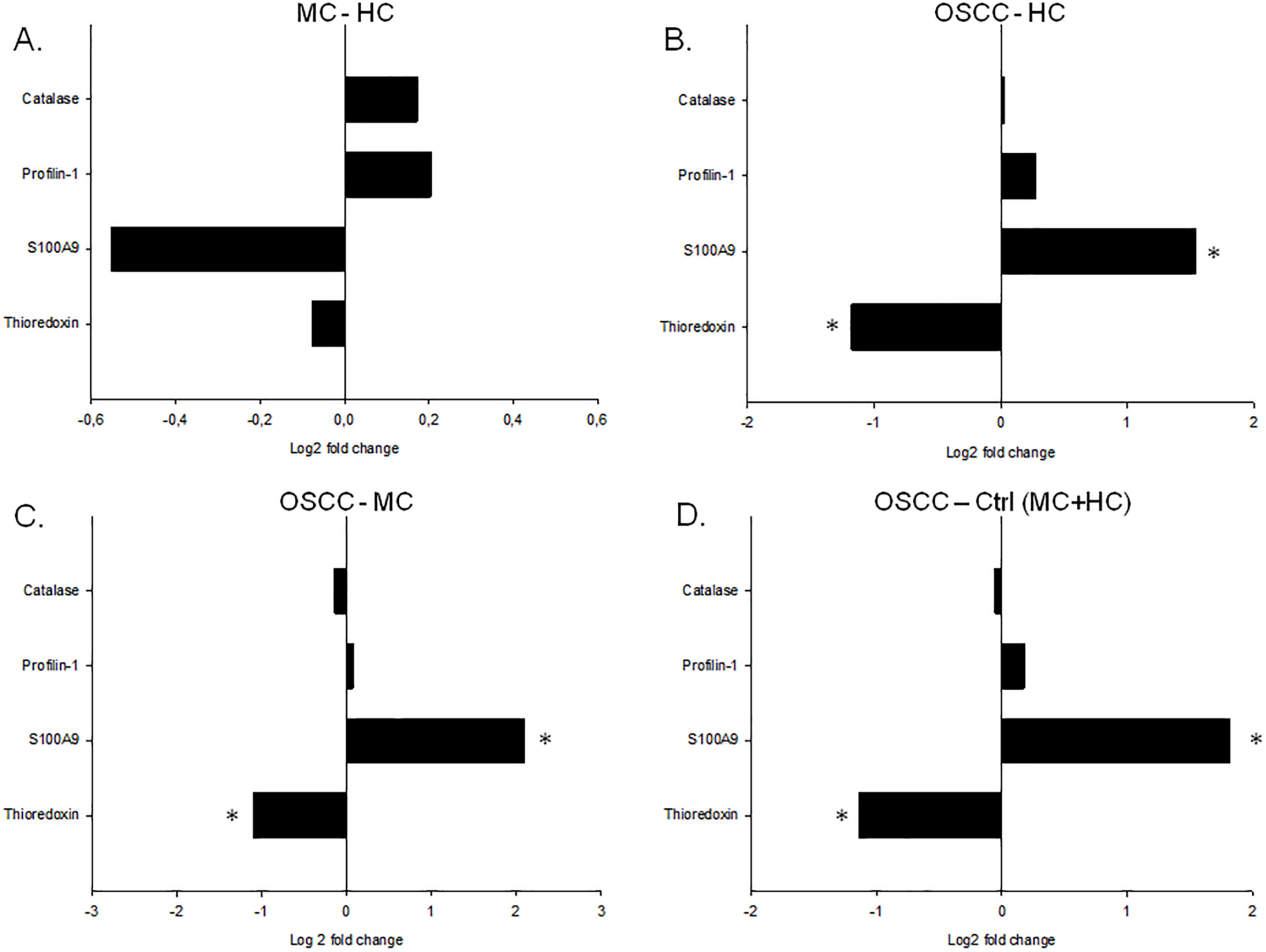
Quantitative analysis of salivary proteins by SRM. The log2 fold change in case of thioredoxin, S100A9, profilin and catalase is shown. The comparison between the (A) age matched controls (MC) and healthy controls (HC), (B) OSCC group and healthy control (HC) group, (C) OSCC group and age matched (MC) group and (D) OSCC group and both healthy and matched control groups is indicated. * indicates significant change, p<0.05.

The statistical analyses indicated no significant differences between age-matched (MC) and healthy young (HC) controls (Fig 3A) while significant differences were observed in case of S100A9 and thioredoxin between patients with OSCC and controls (Fig 3B–3D, Table 4). These data suggest that these changes were specific for OSCC and were not age-dependent. In the saliva of patients with OSCC the level of S100A9 was significantly elevated while the level of thioredoxin was significantly reduced compared to both healthy young and age matched controls. S100A9 is a general marker of cancerous lesions, it was identified in different tumors including tumors of tongue, prostate, breast, lung, in colorectal cancer, gastric cancer, squamous cervical cancer, etc. and it is considered as a primary target for drug development [77–84]. Thioredoxin is part of the antioxidant system and it was shown to have increased expression in tumor samples [85–87]. We do not have information regarding the thioredoxin levels in the salivary gland or tumor tissue itself, but it cannot be excluded that the reduction of thioredoxin levels in saliva may arise from the fact that this protein accumulates in the tissues resulting in a reduced secretion.

**Table 4:** Results of mixed-effect variance analysis of OSCC and MC group differences. The log2 fold change (logFC) standard error (SE), T values (Tvalue) and the FDR corrected p-values are shown.

Protein	Log2FC	SE	Tvalue	Adjusted p-value
Catalase	-0.14	0.25	-0.56	0.66
Profilin-1	0.07	0.17	0.44	0.66
S100A9	2.09	0.41	5.14	<0.0001
Thioredoxin	-1.10	0.39	-2.79	0.01

### ROC analysis

The results of either the mass spectrometry-based or Luminex-based experiments provided information about the changes of the studied proteins in patients with OSCC compared to those in the controls. To test which proteins with a significant difference between the studied groups can be used as possible biomarkers, a ROC curve analysis was performed and the area under the curve (AUC) was calculated. Values of AUC close to 1.0 suggest a perfectly performing biomarker; while values close to 0.5 indicate that the biomarker performs not better than random.

**Fig 4:**
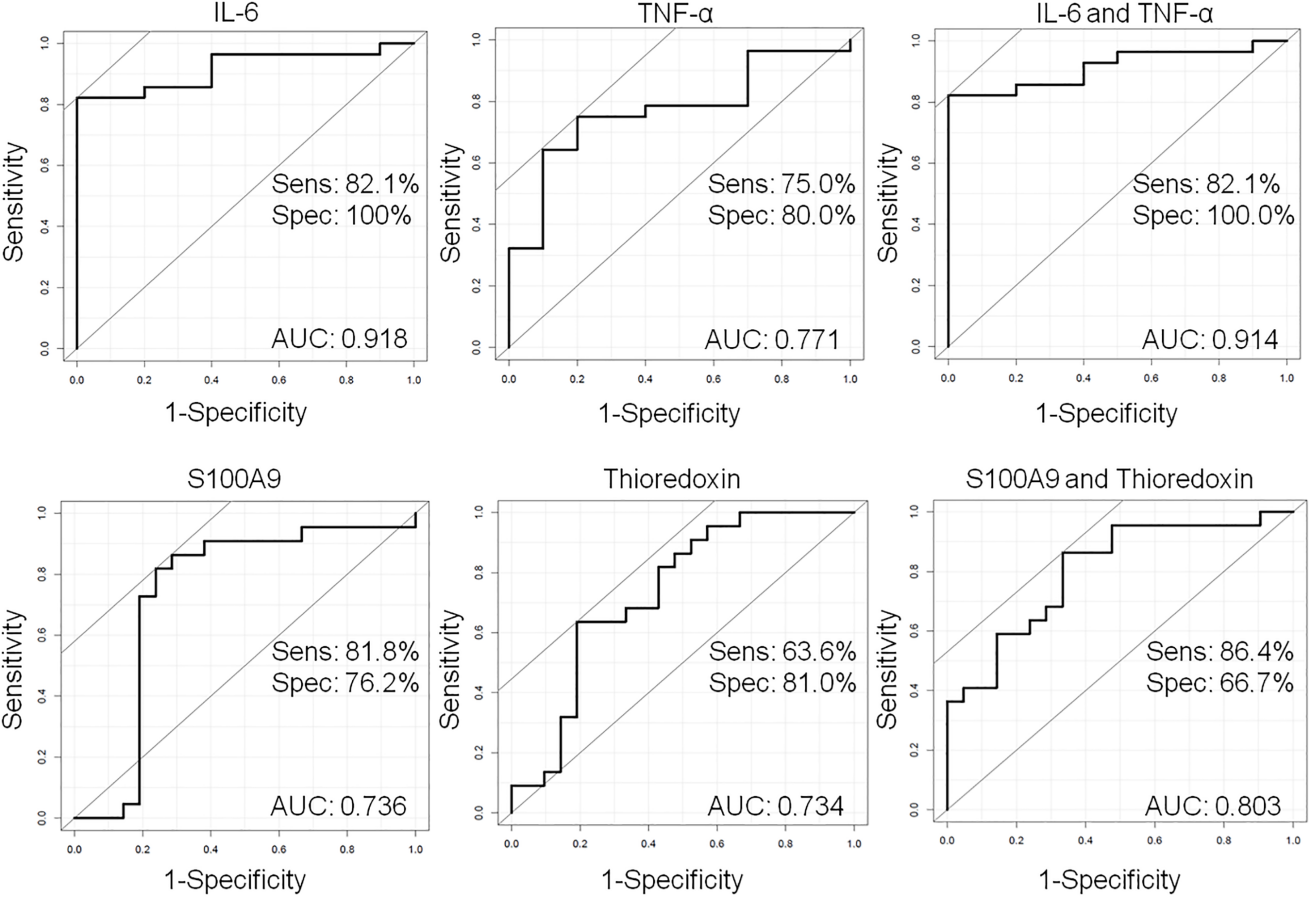
Estimation of predictive power of possible biomarkers using ROC curve analysis. The sensitivity (y axis) was plotted versus 1−specificity (x axis) in case of each potential biomarker alone or in combinations. The area under the curve is indicated on each pane. The sensitivity and specificity was calculated for each biomarkers and biomarker combination.

In our experiments the AUC value for S100A9 was 0.74 (accuracy 0.75, 95% confidence interval: 0.55–0.96) and for thioredoxin 0.73 (accuracy 0.79, 95% confidence interval: 0.61–0.96) suggesting equally performing potential biomarkers (Fig 4). In order to examine the sensitivity and specificity of the combination of S100A9 and thioredoxin multivariate ROC analysis was carried out. The S100A9 and thioredoxin together performed better than alone, the AUC value was 0.80 (accuracy 0.88, 95% confidence interval: 0.77–1.0) suggesting an additive effect of the two potential biomarkers on the discrimination of OSCC samples from the controls (Fig 4).

In the next step we wanted to examine the sensitivity and specificity of the IL-6 and TNF-α alone or in combination. The AUC for IL-6 was 0.92 (accuracy 0.92, 95% confidence interval: 0.83–1.0) and for TNF-α was 0.77 (accuracy 0.77, 95% confidence interval: 0.6–0.93), while the AUC for the combination of IL-6 and TNF-α was 0.91 (accuracy 0.91, 95% confidence interval: 0.83–1.0) (Fig 4). These results indicate that the IL-6 alone seems to be the best potential biomarker able to distinguish between the OSCC samples and controls as it was shown in most of the studies presented in the literature (Table 1).

### Verification of the potential biomarkers using ELISA

**Fig 5:**
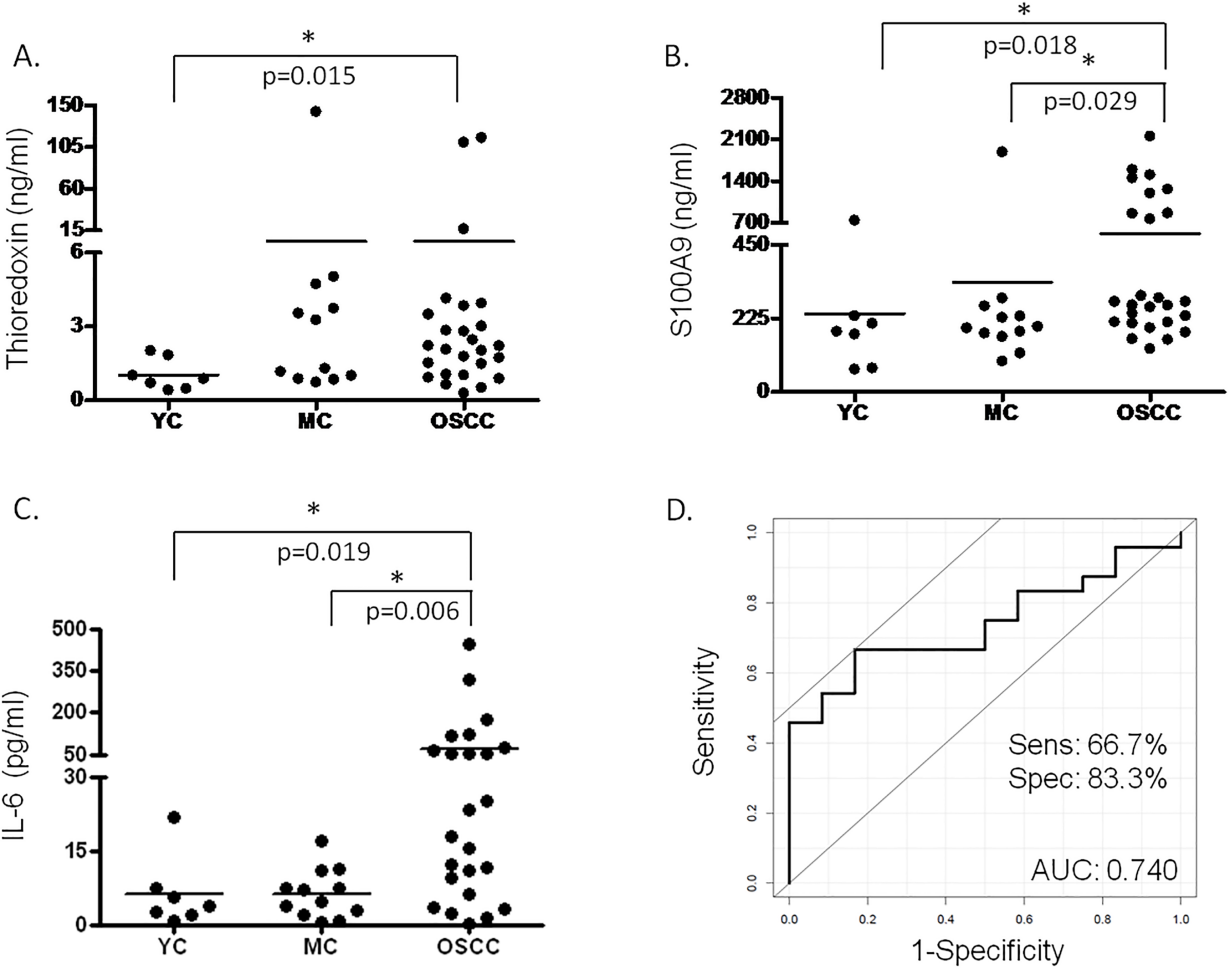
Salivary protein levels determined with ELISA. The salivary concentration of (A) thioredoxin, (B) S100A9 protein and (C) IL-6 is indicated in case of young healthy controls (YC), age matched controls (MC) and OSCC group. In cases where significant difference was observed the p value is indicated. (D) ROC analysis of the combined effect of IL-6 and S100A9 protein.

The level of IL-6, S100A9 and thioredoxin was examined using quantitative ELISA according to the protocol provided by the manufacturers in the saliva samples of patients with OSCC, age-matched controls and young controls. The 46 samples of the reference set were analyzed in duplicates (Fig 5) but for the statistical analysis data for only 45 samples were used (one age-matched control sample was excluded because of missing patient data).

According to the validation experiments in case of IL-6 and S100A9 there was significant difference observed between the OSCC and young controls and OSCC and age-matched controls, while in case of thioredoxin the difference was significant only between OSCC and young controls. ROC analysis was performed for proteins having significantly different levels between the OSCC and age-matched control groups. The AUC for IL-6 was 0.708 with 50% sensitivity and 100% specificity and for S100A9 the AUC was 0.463, the sensitivity was 96% and specificity was 16%. This means that despite the previous good sensitivity and specificity results obtained on the test set, the analyses carried out on the reference set, on a larger sample cohort indicate that either IL-6 or S100A9 alone are not well-enough performing biomarkers. However, their combination with AUC of 0.74, 67% sensitivity and 83% specificity show a well performing biomarker combination. In order to improve the sensitivity and specificity of OSCC detection further research should be done and extension of the analyses on larger populations would be beneficial.

## Conclusion

In this study we developed and optimized an SRM-based targeted proteomic approach for monitoring the level of salivary biomarker candidate proteins and we could identify and verify potential salivary protein biomarkers suitable for the Hungarian population.

Four out of 14 potential salivary protein biomarkers included in our study were shown to have significant differences between the control and OSCC groups and three of them proved worth using as potential biomarkers for the Hungarian population. The validation of IL-6, S100A9 and thioredoxin revealed the potential utility of combination of IL-6 and S100A9.

Our results provide further evidence to literature data demonstrating that general protein biomarkers for OSCC which can be applied world-wide are very hard to find. It should be noted that on protein level there are discrepancies between the different studied groups which highlights the importance of population-tailored proteomics studies to find OSCC-specific protein biomarkers applicable for cost-effective screening purposes. In our opinion our findings are of remarkable importance since the incidence of OSCC is high and shows an increasing tendency not only in the old but in the young generation as well.

## Supporting information

S1 Table List of saliva samples used in the study. Samples in bold were used for both Bioplex and ELISA experiments.
(XLSX)
